# Efficacy and safety of adjunctive corticosteroids in the treatment of severe community-acquired pneumonia: a systematic review and meta-analysis of randomized controlled trials

**DOI:** 10.1186/s13054-023-04561-z

**Published:** 2023-07-08

**Authors:** Jheng-Yan Wu, Ya-Wen Tsai, Wan-Hsuan Hsu, Ting-Hui Liu, Po-Yu Huang, Min-Hsiang Chuang, Mei-Yuan Liu, Chih-Cheng Lai

**Affiliations:** 1 Department of Nutrition, Chi Mei Medical Center, Tainan, Taiwan; 2 Graduate Institute of Medicine, College of Medicine, Kaohsiung Medical University, Kaohsiung, Taiwan; 3 Center of Integrative Medicine, Chi Mei Medical Center, Tainan, Taiwan; 4 Department of Internal Medicine, Chi Mei Medical Center, Tainan, Taiwan; 5 Department of Psychiatry, Chi Mei Medical Center, Tainan, Taiwan; 6 Department of Nutrition and Health Sciences, Chang Jung Christian University, Tainan, Taiwan; 7 Department of Food Nutrition, Chung Hwa University of Medical Technology, Tainan, Taiwan; 8 Division of Hospital Medicine, Department of Internal Medicine, Chi Mei Medical Center, Tainan, Taiwan; 9 School of Medicine, College of Medicine, National Sun Yat-Sen University, Kaohsiung, Taiwan

**Keywords:** Community-acquired pneumonia, Corticosteroid, Mortality, Hydrocortisone, Intensive care unit

## Abstract

**Background:**

This systematic review and meta-analysis aimed to investigate the clinical efficacy and safety of systemic corticosteroids in the treatment of patients with severe community-acquired pneumonia (sCAP).

**Methods:**

A comprehensive search was conducted using the Medline, Embase, ClinicalTrials.gov, and Scopus databases for articles published until April 24, 2023. Only randomized controlled trials (RCTs) that assessed the clinical efficacy and safety of adjunctive corticosteroids for treating sCAP were included. The primary outcome was the 30-day all-cause mortality.

**Results:**

A total of seven RCTs involving 1689 patients were included in this study. Overall, the study group had a lower mortality rate at day 30 than the control group (risk ratio [RR], 0.61; 95% CI 0.44 to 0.85; *p* < 0.01) with low heterogeneity (*I*^2^ = 0%, *p* = 0.42). Compared to the control group, the study group had a lower risk of the requirement of mechanical ventilation (RR 0.57; 95% CI 0.45 to 0.73; *p* < 0.001), shorter length of intensive care unit (MD − 0.8; 95% CI − 1.4 to − 0.1; *p* = 0.02), and hospital stay (MD − 1.1; 95% CI − 2.0 to − 0.1; *p* = 0.04). Finally, no significant difference was observed between the study and the control groups in terms of gastrointestinal tract bleeding (RR 1.03; 95% CI 0.49 to 2.18; *p* = 0.93), healthcare-associated infection (RR 0.89; 95% CI 0.60 to 1.32; *p* = 0.56), and acute kidney injury (RR 0.68; 95% CI 0.21 to 2.26; *p* = 0.53).

**Conclusions:**

In patients with sCAP, adjunctive corticosteroids can provide survival benefits and improve clinical outcomes without increasing adverse events. However, because the pooled evidence remains inconclusive, further studies are required.

**Supplementary Information:**

The online version contains supplementary material available at 10.1186/s13054-023-04561-z.

## Introduction

Severe community-acquired pneumonia (sCAP) is a leading cause of hospitalization and can result in significant morbidity and mortality, particularly among vulnerable populations such as elderly, immunocompromised individuals, and those with chronic medical conditions [1, 2]. Despite developments in antimicrobial therapy and life-support measures for patients with sCAP, clinical outcomes have not improved significantly owing to an aging population, increased prevalence of comorbidities, and the emergence of multi-drug-resistant organisms. Therefore, the prevention and effective management of sCAP continue to be critical public health concerns [3].

In addition to early diagnosis and appropriate antimicrobial therapy, the use of corticosteroids for the management of sCAP has been discussed in considerable detail [4]. As numerous randomized controlled trials (RCTs) have investigated the role of adjunctive corticosteroids in the treatment of sCAP and yielded inconsistent findings [5–10], current guidelines provide different recommendations regarding the use of corticosteroids in patients with sCAP [11, 12]. Furthermore, several systematic reviews and meta-analyses have explored the efficacy of corticosteroids in the treatment of patients with CAP; however, not all studies included in these meta-analyses focused on sCAP, and no consistent findings have been reported [13–16]. In 2023, a large randomized controlled trial (RCT) reported that among patients with sCAP treated in the intensive care unit (ICU), treatment with hydrocortisone could result in a lower risk of 28-day mortality than those treated by a placebo [17]. To address this controversy, we conducted an updated systematic review and meta-analysis of RCTs to evaluate the clinical effectiveness and safety of adjunctive corticosteroid therapy in patients with sCAP.

## Methods

This systematic review and meta-analysis adhered to the reporting guidelines of Preferred Reporting Items for Systematic Reviews and Meta-analyses (PRISMA) [18]. The study protocol was registered with PROSPERO.

### Search strategy and study selection

We performed a comprehensive systematic search of the Medline, Embase, ClinicalTrials.gov, and Scopus databases for articles published between the database inception and April 24, 2023, using appropriate prespecified search terms: “community-acquired pneumonia,” “corticosteroid,” and “steroid.” Only RCTs that assessed the clinical efficacy and safety of systemic corticosteroids in the treatment of adult patients with sCAP were included. To identify relevant reports, we manually searched the reference lists of systematic and narrative reviews and studies that fulfilled the eligibility criteria of the present study.

### Inclusion and exclusion criteria

Studies were included if the PICO (population, intervention, comparison, outcomes) criteria were met: (a) Population: adult patients (i.e., 18 years of age or older) with sCAP; (b) Intervention: systemic corticosteroids were used regardless of the type of corticosteroid, duration of treatment, dosage, or route of administration (i.e., intervention group); (c) Comparison: placebo or standard care (i.e., control group), (d) Outcomes: mortality, the use of mechanical ventilation (MV), length of intensive care unit ICU stay, length of hospital stay, and the adverse events (AEs). In this study, sCAP was defined as CAP accompanied by requiring ICU admission, or meeting either the criteria for severe pneumonia by the American Thoracic Society/Infectious Diseases Society of America (ATS/IDSA) [19] or classified as risk class V of the Pneumonia Severity Index (PSI) [20]. Only peer-reviewed RCTs were included in the analysis without restrictions on language, sample size, age, sex, ethnicity, or publication date.

We excluded studies that (1) focused on patients with septic shock; (2) reported data from post-hoc analysis; (3) were published only as conference posters, case series, case reports, or single-arm studies; (4) did not report outcomes of interest; or (5) were pharmacokinetic investigations.

### Study selection

Two independent investigators (JYW and YWT) screened the titles and abstracts of the studies to identify potentially eligible studies. Full-text copies of the potentially relevant articles were obtained and reviewed for eligibility. In case of disagreement, a third investigator (WHH) was consulted.

### Data extraction

Two investigators (JYW and YWT) independently extracted information, such as author name, year of publication, study sites and country, age, and sex of the study participants, sample size, and systemic corticosteroid regimens from the included RCTs. In the case of discrepancies, a third reviewer (WHH) was consulted to make the final decision regarding the data collection process.

### Outcome and definitions

The primary outcome was 30-day all-cause mortality, while the secondary outcomes included MV requirement, length of ICU stay, length of hospital stay, and AEs including gastrointestinal (GI) tract bleeding, healthcare-associated infection (HAI), acute kidney injury (AKI), and hospital readmission. Prespecified subgroup analyses focusing on mortality were performed based on the regimens of systemic corticosteroids, age, use of MV, the status of septic shock on enrollment, and ICU admission upon randomization.

### Assessment of risk of bias

The revised Cochrane risk-of-bias tool 2.0 [19] was used to assess the quality of each included study [21]. Two of the investigators (THL and PYH) independently reviewed all included studies and rated them as having “low risk,” “some concerns,” or “high risk” of bias based on the following domains: randomization process, deviations from intended interventions, missing outcome data, measurement of outcome, and selection of reported result. In case of disagreement, a third investigator (JYW) was consulted, and a consensus was reached through discussion.

### Statistical analysis

We calculated the risk ratio (RR) and 95% confidence intervals (CIs) to estimate binary variables and the mean difference with a 95% CI for continuous variables. Heterogeneity was estimated using the *I*^2^ statistic, and significance was defined as *I*^2^ above 50%. For the primary outcome, we conducted a leave-one-out sensitivity analysis to assess the effects of individual studies on the overall outcomes. All analyses were performed using Review Manager (RevMan) version 5.4.1 (The Nordic Cochrane Centre, The Cochrane Collaboration, Copenhagen, 2014). We conducted two-tailed tests for all comparisons, and statistical significance was defined as a *p*-value less than 0.05.

We utilized the R package “meta” and performed a Mantel–Haenszel and Inverse Variance-weighted random-effects model to estimate the overall effect. All *p*-values were calculated using a two-tailed test and considered statistically significant if they were less than 0.05, except for the determination of the statistical test for heterogeneity, which used a threshold of 0.1.

### Trial sequential analysis (TSA)

We performed TSA [22] to assess the reliability of the cumulative evidence. To calculate the required information size (RIS) for primary and secondary outcomes, we used a type I error of 5%, power of 80%, and reduction in relative risk of 20%. We then examined the association of the cumulative *Z*-curve with the TSA boundary or RIS to determine the strength of the evidence.

## Results

### Study selection

**Fig. 1:**
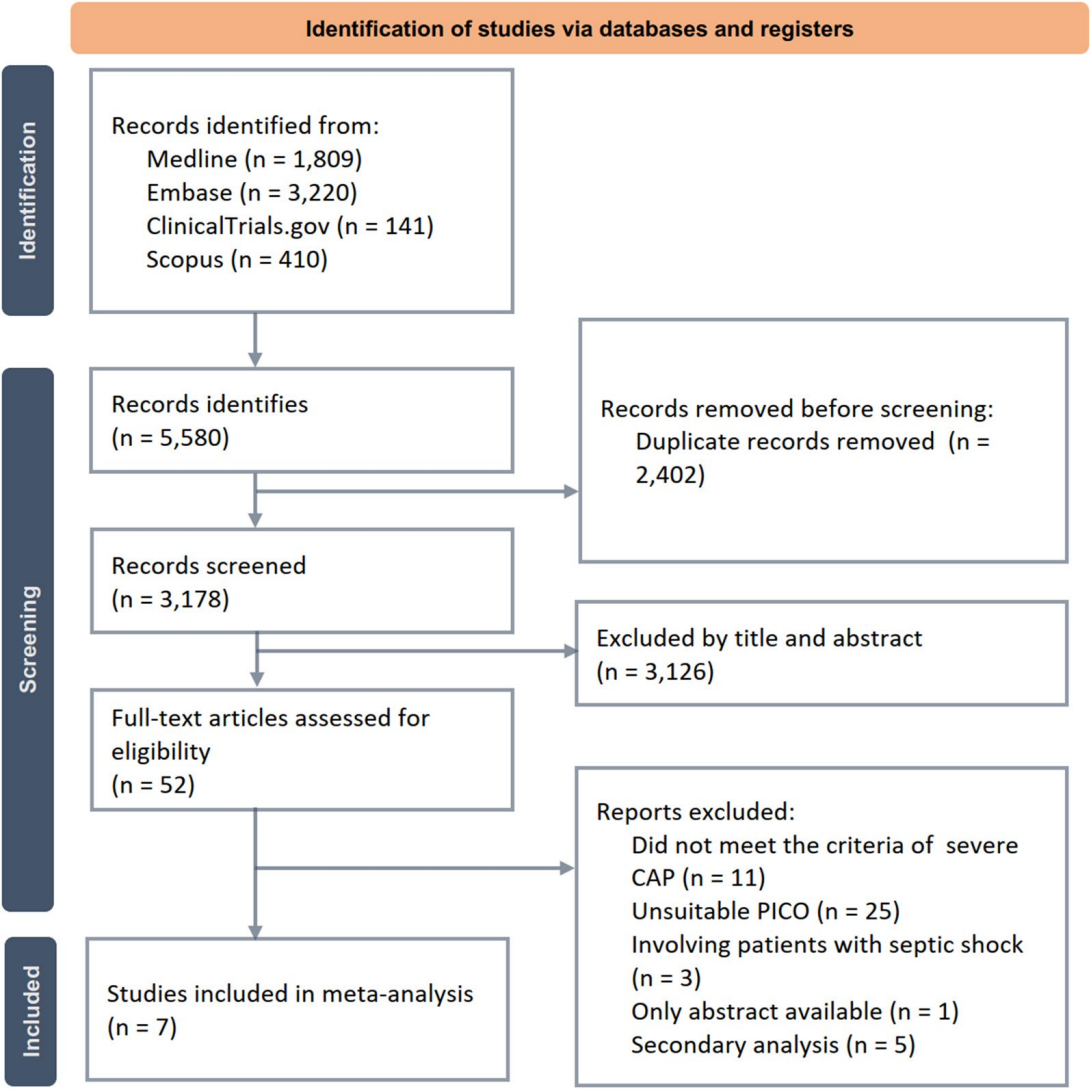
Algorithm of study selection

First, we identified 5580 records from the Medline (*n* = 1809), Embase (*n* = 3220), ClinicalTrials.gov (*n* = 141), and Scopus databases (*n* = 410). After removing 2402 duplicate records and 3126 irrelevant articles based on titles and abstracts, 52 reports were screened for eligibility. After excluding 45 studies that did not meet the selection criteria, we identified seven studies [5–10, 17]. The process for selecting studies is outlined in Fig. 1.

**Fig. 2:**
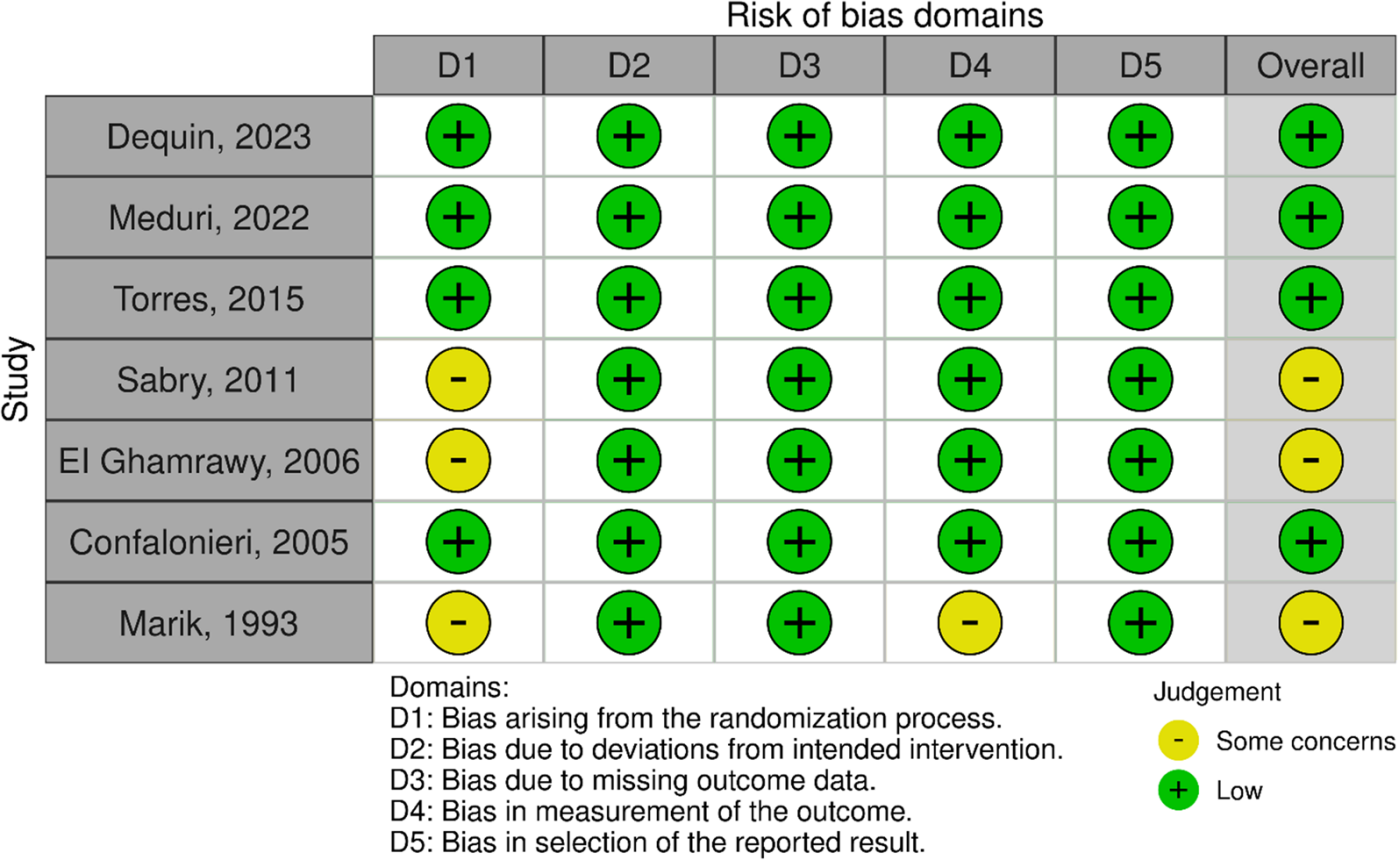
Assessment of risk of bias

Figure 2 shows the assessment of risk of bias. Three RCTs [5, 7, 10] had the unclear risk of bias in the randomization process, and one RCT [5] had unclear risk of bias in the measurement of the outcomes. Other four RCTs [6, 8, 9, 17] had low risk of bias in all domains.

### Characteristics of included studies

This meta-analysis included seven double-blind RCTs [5–10, 17] (Table 1), in which five [6–9, 17] were multicenter RCTs. Two of the RCTs were conducted in the US [5, 9], while the others were conducted in Spain [8], Saudi Arabia [10], Italy [6], France [17], and Egypt [7]. Six studies [5–7, 9, 10, 17] included only patients with sCAP who required ICU admission.

**Table 1:** Characteristics of included studies

Study	Design	Study site	Patients	Regimen of corticosteroid	No. of patients	
					Study	Control
Dequin et al. [17]	Double-blind, randomized, controlled trial	31 centers in French	Adult patients with severe CAP requiring ICU admission	Hydrocortisone, 200 mg daily for either 4 or 8 days	400	395
Meduri et al. [9]	Double-blind, randomized, placebo-controlled clinical trial	42 centers in US	Adult patients with severe CAP requiring ICU admission	Methylprednisolone 40 mg loading followed by 40 mg/day through day 7 and progressive tapering for 20 days	297	287
Torres et al. [8]	Double-blind, randomized, placebo-controlled clinical trial	3 centers in Spain	Adult patients with severe CAP	Methylprednisolone 0.5 mg/kg q12h for 5 days	61	59
Sabry et al. [7]	Double-blind, randomized, controlled trial	2 centers in Egypt	Adult patients with severe CAP requiring ICU admission	Hydrocortisone, loading dose of 200 mg, followed by 300 mg daily for 7 days	40	40
El-Ghamrawy et al. [10]	Double-blind, randomized, controlled trial	Saudi Arabia	Adult patients with severe CAP requiring ICU admission	Hydrocortisone, loading dose of 200 mg, followed by 240 mg daily for 7 days	17	17
Confalonier et al. [6]	Double-blind, randomized, placebo-controlled clinical trial	6 centers in Italy	Adult patients with severe CAP requiring ICU admission	Hydrocortisone, 200-mg bolus followed by infusion at a rate of 10 mg/hour for 7 days	23	23
Marik et al. [5]	Double-blind, randomized, placebo-controlled clinical trial	1 center in US	Adult patients with severe CAP requiring ICU admission	Hydrocortisone 10 mg/kg once	14	16

CAP, community-acquired pneumonia; ICU, intensive care unit

A total of 1689 patients were included in this meta-analysis, of whom 852 were classified as the study group receiving systemic corticosteroids and 837 as the control group who did not receive corticosteroids. The corticosteroids tested in these trials were hydrocortisone (*n* = 5 [5–7, 10, 17]), and methylprednisolone (*n* = 2) [8, 9]. Except for one study that used a single dose of hydrocortisone [5], the other six studies used systemic corticosteroids for at least four days [6–10, 17]. The mean or median age of the patients in five trials [6–9, 17] was more than 60 years, and male patients were predominant [6–9, 17]. In three trials, more than 40% of patients required MV [6, 7, 17](Table 2).

**Table 2:** Demographic features of included patients

Study	Age, years		Male sex, No. (%)		Disease severity		Mechanical ventilation, No. (%)		ICU admission, No. (%)	
	Study group	Control group	Study group	Control group	Study group	Control group	Study group	Control group	Study group	Control group
Dequin et al. [17]	67 (58–77)	67 (58–78)	281 (70)	271 (69)	SOFA: 4 (3–6) PSI V: 181 (46)	SOFA: 4 (3–6) PSI V: 193 (49)	178 (45)	175 (44)	400 (100)	395 (100)
Meduri et al. [9]	69 ± 11	69 ± 11	289 (97)	273 (95)	PSI V: 119 (40) APACHE III: 54.3 ± 29.4	PSI V: 113 (40) APACHE III: 53.4 ± 28.7	97 (33)	96 (33)	NA	NA
Torres et al. [8]	65 ± 19	66 ± 20	35 (57)	39 (66)	PSI V: 22 (36)	PSI V: 19 (32)	1 (2)	2 (3)	43 (70)	47 (80)
Sabry et al. [7]	62 ± 7	63 ± 4	30 (75)	28 (70)	SOFA: 8.5 ± 1.5	SOFA: 8.2 ± 1.5	26 (65)	34 (85)	40 (100)	40 (100)
El-Ghamrawy et al. [10]	NA	NA	NA	NA	NA	NA	NA	NA	NA	NA
Confalonier et al. [6]	60 ± 17	67 ± 15	17 (74)	15 (65)	APACHE II: 17.2 ± 4.1	APACHE II: 18.2 ± 4.0	15 (65)	19 (83)	23 (100)	23 (100)
Marik et al. [5]	32 ± 13	40 ± 17	NA	NA	APACHE II: 11 ± 2	APACHE II: 14 ± 6	0 (0)	0 (0)	14 (100)	16 (100)

MV, mechanical ventilation; APACHE, Acute Physiology and Chronic Health; PSI, pneumonia severity index; ICU, intensive care unit; SOFA, Sequential Organ Failure Assessment; NA, not applicable

### Primary outcomes

**Fig. 3:**
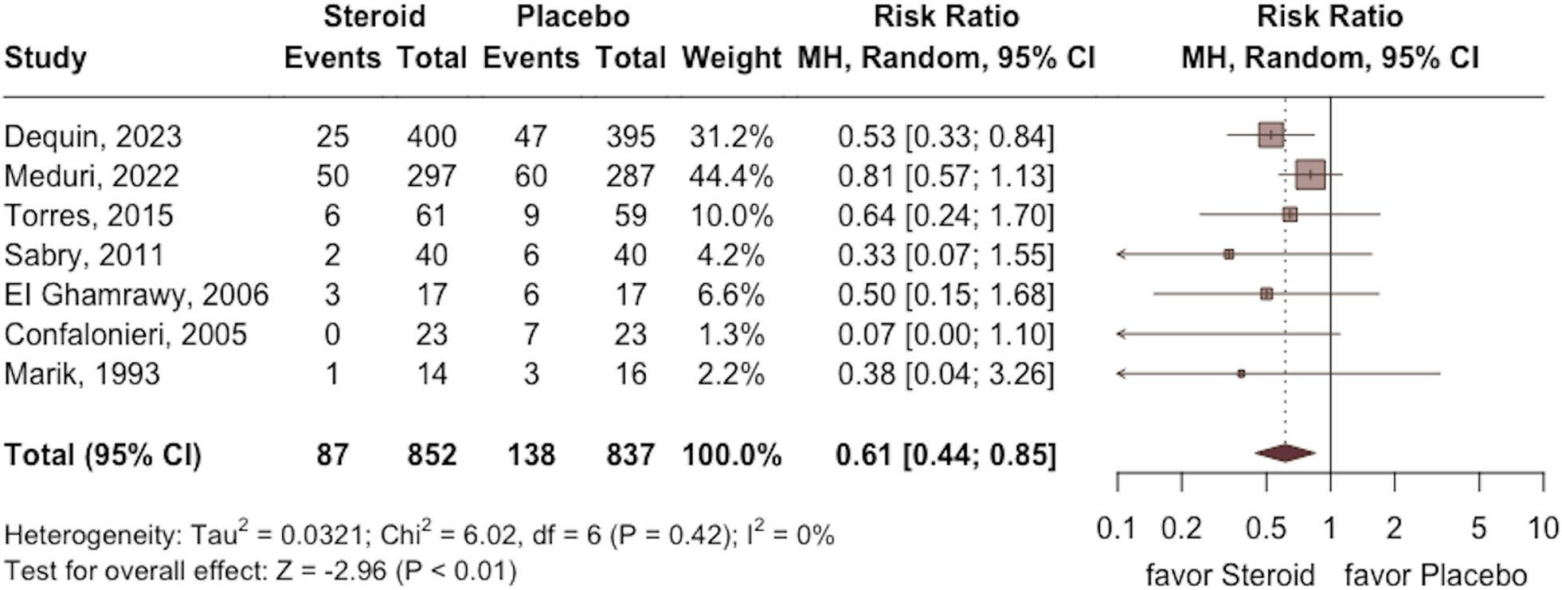
Forest plot comparing all-cause mortality between the study group receiving corticosteroids and the control group without corticosteroids

Overall, the study group had a lower mortality rate at day 30 than the control group (RR 0.61; 95% CI 0.44 to 0.85; *p* < 0.01; seven RCTs, 1689 participants, Fig. 3) with low heterogeneity (*I*^2^ = 0%, *p* = 0.42). The significant difference in mortality between the study and control groups remained unchanged in the leave-one-out sensitivity test, in which individual studies were randomly excluded (Additional file 1: Fig. S1). Further subgroup analyses consistently revealed a lower mortality rate in the study group compared to the control group. However, the differences remained statistically significant only within specific patient subgroups, which included those aged 60 years or older, without septic shock on enrollment, with ICU admission, use of hydrocortisone, and receiving corticosteroid for a duration of ≤ eight days and not undergoing corticosteroid tapering (Table 3).

**Table 3:** Subgroup analysis of all-cause mortality

Subgroup	No. of study	No. of participant	Risk ratio	95% CI	*I*^2^ (%)
Patients aged ≥ 60 years	6	1659	0.62	0.45 to 0.86	16
*Septic shock status on enrollment*					
Presence (≤ 50%)	5	1625	0.61	0.42 to 0.90	30
Absence	1	795	0.53	0.33 to 0.84	
Use of mechanical ventilation upon randomization	2	546	0.95	0.59 to 1.53	31
*Definition of sCAP*					
ICU admission on randomization	6	1569	0.60	0.42 to 0.86	19
Meet ATS/IDSA criteria or PSI V	4	830	0.62	0.34 to 1.11	31
*Type of corticosteroid*					
Hydrocortisone	5	985	0.48	0.30 to 0.72	0
Methylprednisolone	2	704	0.79	0.57 to 1.08	0
*Corticosteroid tapering*					
No	6	1105	0.50	0.34 to 0.73	0
Yes	1	584	0.81	0.58 to 1.13	
*Duration of corticosteroid treatment*					
≤ 8 days	6	1105	0.50	0.34 to 0.73	0
> 8 days	1	584	0.81	0.58 to 1.13	

sCAP, severe community-acquired pneumonia; ICU, intensive care unit; ATS/IDSA, American Thoracic Society/Infectious Diseases Society of America; PSI, pneumonia severity index

### Secondary outcome

**Fig. 4:**
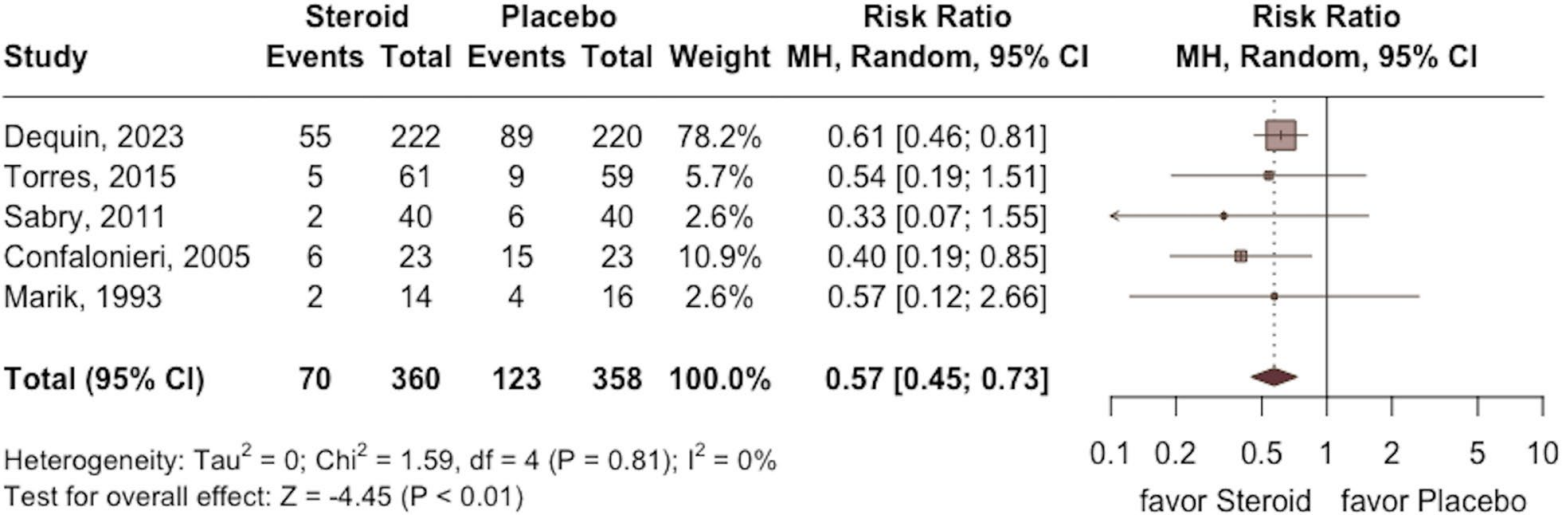
Forest plot comparing the risk of mechanical ventilation between the study group receiving corticosteroids and the control group without corticosteroids

**Fig. 5:**
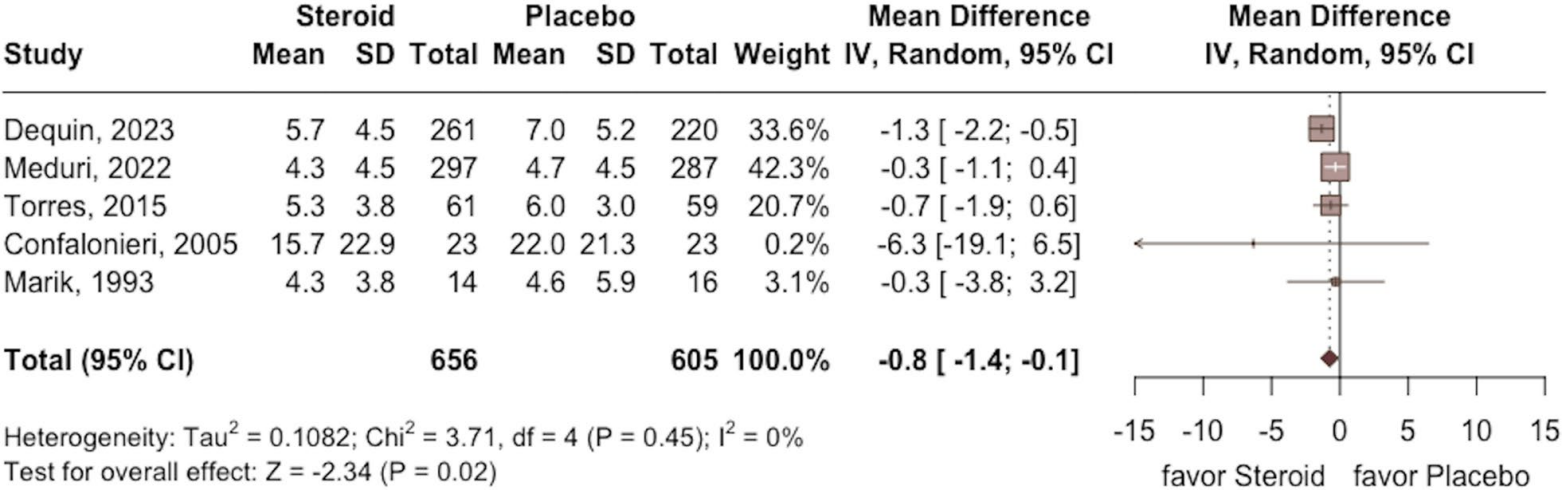
Forest plot comparing the length of intensive care unit stay between the study group receiving corticosteroids and the control group without corticosteroids

**Fig. 6:**
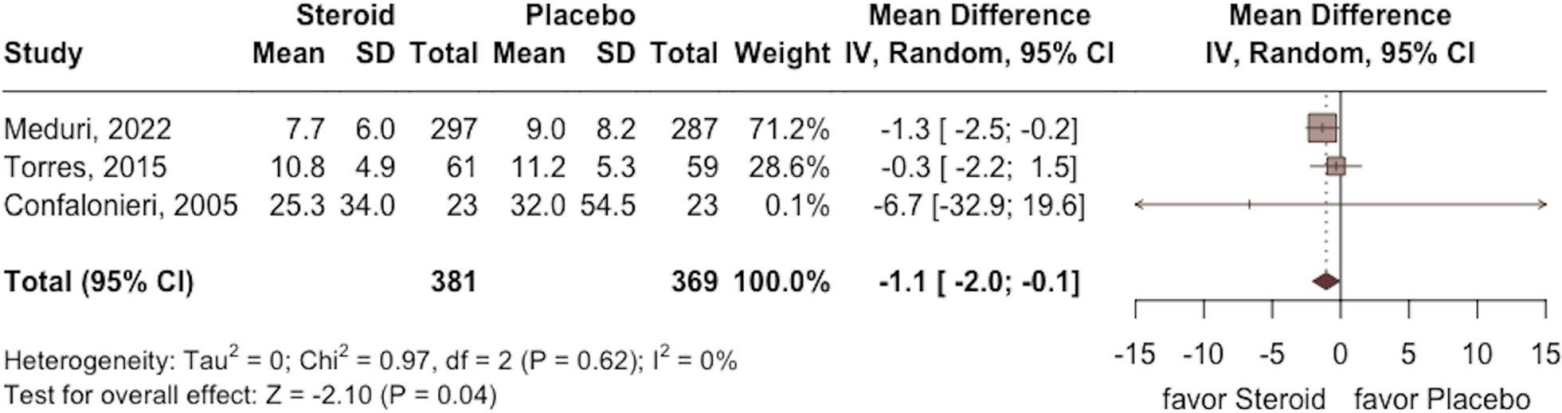
Forest plot comparing the length of hospital stay between the study group receiving corticosteroids and the control group without corticosteroids

The requirement of MV was lower in the study group than in the control group (RR 0.57; 95% CI 0.45 to 0.73; *p* < 0.001, five RCTs, 718 participants: Fig. 4) with low heterogeneity (*I*^2^ = 0%, *p* = 0.81). In addition, a shorter length of ICU and hospital stay were observed in the study group compared with the control group (ICU stay: MD − 0.8; 95% CI − 1.4 to − 0.1; *p* = 0.02; five RCTs, 1261 participants; Fig. 5; hospital stay: MD − 1.1; 95% CI − 2.0 to − 0.1; *p* = 0.04; three RCTs, 750 participants; Fig. 6) based on low heterogeneity (ICU stay: *I*^2^ = 0%, *p* = 0.45; hospital stay: *I*^2^ = 0%, *p* = 0.62).

**Fig. 7:**
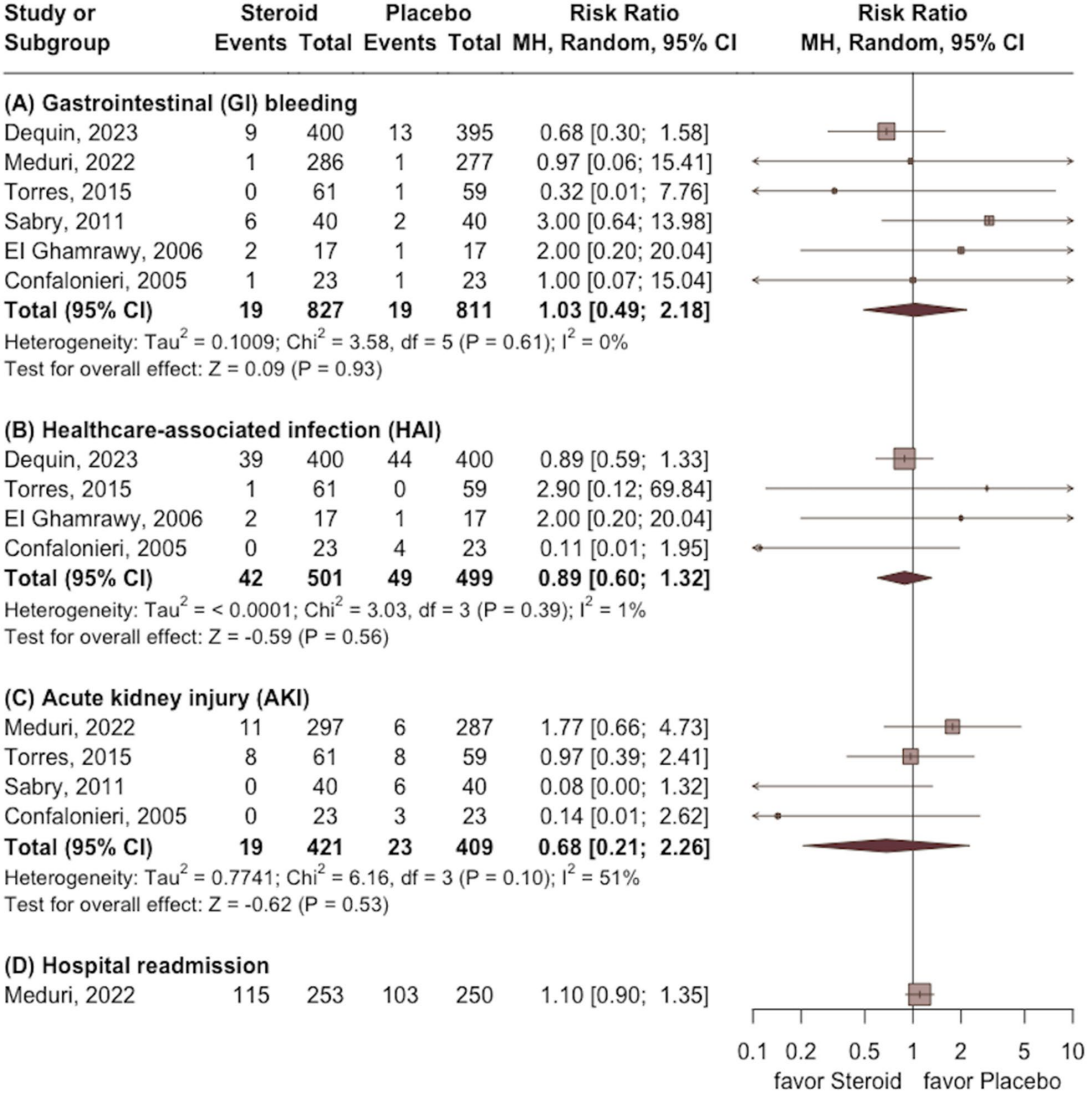
Forest plot comparing the risk of gastrointestinal (GI) bleeding, healthcare-associated infection (HAI), acute kidney injury (AKI), and hospital readmission between the study group receiving corticosteroids and the control group without corticosteroids

Regarding AE, no significant difference was observed between the study and the control groups in terms of GI tract bleeding (RR 1.03; 95% CI 0.49 to 2.18; *p* = 0.93), HAI (RR 0.89; 95% CI 0.60 to 1.32; *p* = 0.56), AKI (RR 0.68; 95% CI 0.21 to 2.26; *p* = 0.53), and hospital readmission (RR 1.10; 95% CI 0.90 to 1.35) (Fig. 7).

### Trial sequence analysis

The results of the TSA analysis on the 30-day all-cause mortality were inconclusive, indicating that the Z-curve did not cross the benefit boundary (Additional file 1: Fig. S2). Furthermore, the current analysis only accounted for 39.4% of the RIS cases (1689/4286 patients). However, the TSA analysis provided robust evidence for a true-positive result in the risk of mechanical ventilation, length of ICU, and hospital stay (Additional file 1: Figs. S3–S5 ). In terms of AEs, the *Z*-curves failed to reach the traditional boundaries, or the TSA boundaries, and inner wedge (Additional file 1: Figs. S6–S8).

## Discussion

This meta-analysis investigated the clinical efficacy and safety of adjunctive corticosteroids in the treatment of patients with sCAP and demonstrated that systemic corticosteroids are associated with a better clinical outcome. First, based on the analysis of seven RCTs [5–10, 17], additional corticosteroids for treating critically ill patients with sCAP were associated with a significantly lower mortality rate than placebo or usual care alone. A similar trend was observed in the leave-one-out sensitivity test. Secondly, lower mortality among sCAP patients receiving corticosteroids compared to the control group was consistently observed in most of the subgroup analyses, particularly for patients those aged 60 years or older, without initial septic shock, with ICU admission, use of hydrocortisone, and receiving corticosteroid for a duration of ≤ eight days and not undergoing corticosteroid tapering. Finally, patients receiving systemic corticosteroids had a lower risk of requiring further MV and shorter ICU and hospital stays than the control group. These findings indicate that adjunctive corticosteroids can improve the clinical outcomes of patients with sCAP and support their use in this clinical setting. Despite the guidelines recommending the use of corticosteroids in sCAP patients with septic shock [23, 24], the majority of patients included in this meta-analysis did not have initial septic shock. The analysis demonstrated a beneficial effect of adjunctive corticosteroids in these patients (Table 3). This finding further suggests that corticosteroids may have a role in the treatment of sCAP even in the absence of initial septic shock.

In contrast with our results, previous meta-analyses reported that adding systemic corticosteroids to the treatment of patients with CAP does not significantly affect mortality [15, 25]. Saleem et al. conducted a meta-analysis of 16 RCTs and found no significant difference in mortality between patients receiving adjuvant corticosteroid therapy compared with standard care (9.5% vs 10.8%; RR 0.85; 95% CI 0.67 to 1.07]; *p* = 0.17; *I*^2^ = 14%). Similarly, Briel et al. conducted a meta-analysis based on individual patient data and found no significant difference in 30-day all-cause mortality between patients receiving corticosteroids and those receiving a placebo (5.0% versus 5.9%; adjusted odds ratio: 0.75; 95% CI 0.46 to 1.21; *p* = 0.24) with significant heterogeneity in treatment effects across the included trials (*p* = 0.046 by likelihood ratio test) [25]. The difference between our study and previous meta-analyses could be due to the inclusion of both non-severe and severe CAPs in their analyses [15, 25]; however, we only included patients with sCAP in our study. All these findings indicate that the effect of additional corticosteroids for treating CAP may differ according to disease severity and suggest that adjunctive corticosteroids can provide survival benefits only for severe CAP.

In addition to the clinical benefit of systemic corticosteroids in the treatment of sCAP, clinicians should be cautious about the potential harms, including hyperglycemia, myopathy, superinfection, osteopenia, GI bleeding, weight gain, and brushing [26]. In this study, the AEs such as GI bleeding, HAI, AKI, and hospital readmission were evaluated, and it was found that the use of systemic corticosteroids did not increase these AEs. These findings are consistent with previous studies [15, 25]. Although hyperglycemia is a common adverse effect associated with systemic corticosteroid use, other corticosteroid-related AEs such as nosocomial infections, empyema, GI bleeding, and neuropsychiatric complications are rare and occur at a similar rate of incidence in both the corticosteroid and placebo groups in the treatment of CAP [15, 25]. Overall, these findings suggest that systemic corticosteroids are generally safe for use in the treatment of sCAP; however, glucose levels should be closely monitored and appropriate management strategies should be implemented to mitigate the risk of hyperglycemia.

The current meta-analysis has several strengths. First, we focused only on sCAP to avoid the confounding effects of disease severity. In addition, the low heterogeneity in most outcomes, which may be attributed to similar infection types and patient characteristics among the included studies, may indicate a low risk of bias. Second, despite the need for further evidence to elucidate the survival benefit of corticosteroids on TSA, there was robust evidence to support a lower risk of MV and shorter length of stay in the intervention group. Therefore, our findings suggest clinical benefits of adjunctive corticosteroids in the treatment of patients with sCAP.

This study has several limitations. First, the number of studies focusing on the adjunctive use of corticosteroids in patients with sCAP is limited, which may contribute to the inconclusive evidence regarding the survival benefit and safety of TSA. Secondly, it is noteworthy that the definitions of sCAP employed in each included RCTs were not the same. However, our subgroup analysis of these variations displayed a similar trend. Lastly, the dose, regimen, and treatment duration of corticosteroids varied among the included RCTs, making it difficult to determine the optimal use of corticosteroids in this clinical context. In this study, we found patients who received hydrocortisone and who used corticosteroids with a duration of ≤ eight days and without tapering had significantly lower risks of mortality. These results suggested that the optimal corticosteroid regimen for sCAP might be hydrocortisone with a treatment duration of ≤ eight days and without tapering. However, further study is warranted to clarify these issues.

In conclusion, the current meta-analysis showed that systemic corticosteroids can provide additional survival and other clinical benefits, including a lower risk of MV use and shorter ICU and hospital stays in the treatment of patients with sCAP. In addition, adjunctive corticosteroids did not increase the AEs such as GI tract bleeding, HIA, and AKI in this clinical entity. However, inconclusive evidence was found from trial sequential analysis, and further studies are warranted to verify our findings.

### Supplementary Information

**Additional file 1. Figure S1** Sensitivity analysis of primary outcome by excluding each study individually. **Figure S2** Trial sequence analysis on 30-day all-cause mortality. **Figure S3** Trial sequence analysis on the risk of mechanical ventilation. **Figure S4** Trial sequence analysis on length of intensive care unit stay. **Figure S5** Trial sequence analysis on the length of hospital stay. **Figure S6** Trial sequence analysis on risk of gastrointestinal tract bleeding. **Figure S7** Trial sequence analysis on risk of healthcare-associated infection. **Figure S8** Trial sequence analysis on risk of acute kidney injury

## Data Availability

The datasets used and/or analyzed during the current study are available from the corresponding author on reasonable request.
