# Correction to: Efficacy and safety of adjunctive corticosteroids in the treatment of severe community‑acquired pneumonia: a systematic review and meta‑analysis of randomized controlled trials

**DOI:** 10.1186/s13054-023-04710-4

**Published:** 2023-10-27

**Authors:** Jheng‑Yan Wu, Ya‑Wen Tsai, Wan‑Hsuan Hsu, Ting‑Hui Liu, Po‑Yu Huang, Min‑Hsiang Chuang, Mei‑Yuan Liu, Chih‑Cheng Lai

**Affiliations:** 1 Department of Nutrition, Chi Mei Medical Center, Tainan, Taiwan; 2 Graduate Institute of Medicine, College of Medicine, Kaohsiung Medical University, Kaohsiung, Taiwan; 3 Center of Integrative Medicine, Chi Mei Medical Center, Tainan, Taiwan; 4 Department of Internal Medicine, Chi Mei Medical Center, Tainan, Taiwan; 5 Department of Psychiatry, Chi Mei Medical Center, Tainan, Taiwan; 6 Department of Nutrition and Health Sciences, Chang Jung Christian University, Tainan, Taiwan; 7 Department of Food Nutrition, Chung Hwa University of Medical Technology, Tainan, Taiwan; 8 Division of Hospital Medicine, Department of Internal Medicine, Chi Mei Medical Center, Tainan, Taiwan; 9 School of Medicine, College of Medicine, National Sun Yat-Sen University, Kaohsiung, Taiwan

**Correction to:** **Crit Care 27, 274 (2023). ****https://doi.org/10.1186/s13054-023-04561-z**

Following publication of the original article [1], the authors identified an error within the first sentence of the Results section in the Abstract.

The Results section currently reads:

**Results**

A total of severe RCTs involving 1689 patients were included in this study.

The Results section should read:

**Results**

A total of seven RCTs involving 1689 patients were included in this study.

The Results section has been updated in this correction and the original article [1] has been corrected.

## References

[1] Wu JY, Tsai YW, Hsu WH (2023). Efficacy and safety of adjunctive corticosteroids in the treatment of severe community-acquired pneumonia: a systematic review and meta-analysis of randomized controlled trials. Crit Care.

