# Supplementary material for: Efficacy and safety of adjunctive corticosteroids in the treatment of severe community-acquired pneumonia: a systematic review and meta-analysis of randomized controlled trials
Source: Crit Care. 2023 Jul 8;27:274. doi: 10.1186/s13054-023-04561-z (PMC10329791; doi:10.1186/s13054-023-04561-z)

Supplemental Figure 1. Sensitivity analysis of primary outcome by excluding each study individually


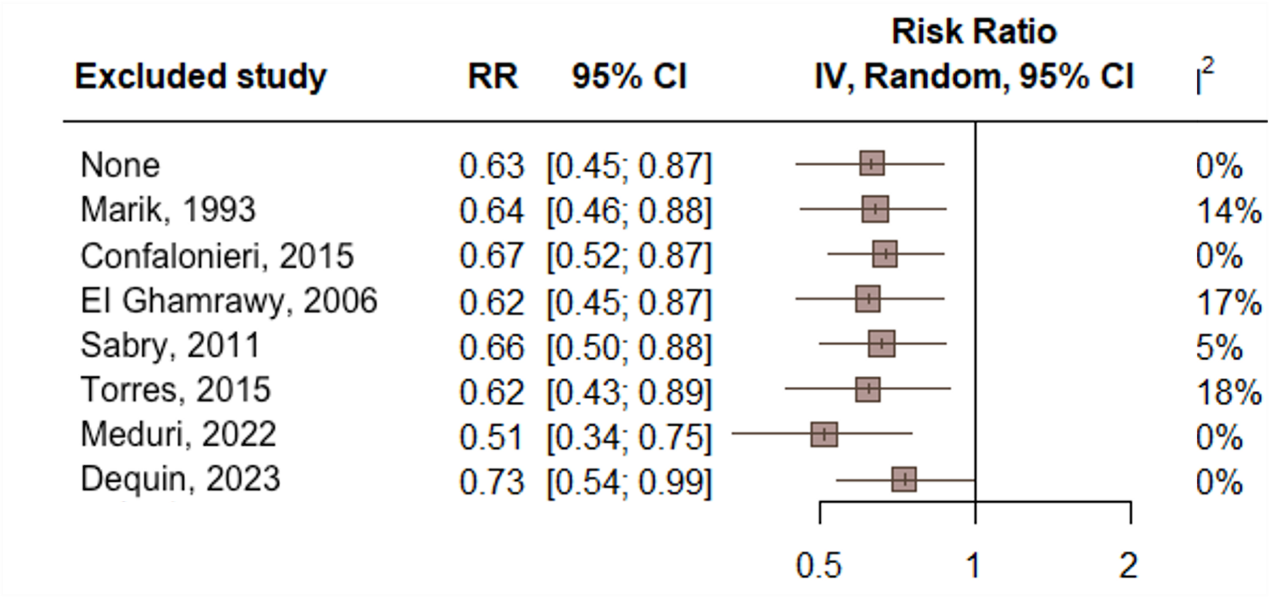


Supplemental Figure 2. Trial sequence analysis on 30-day all-cause mortality


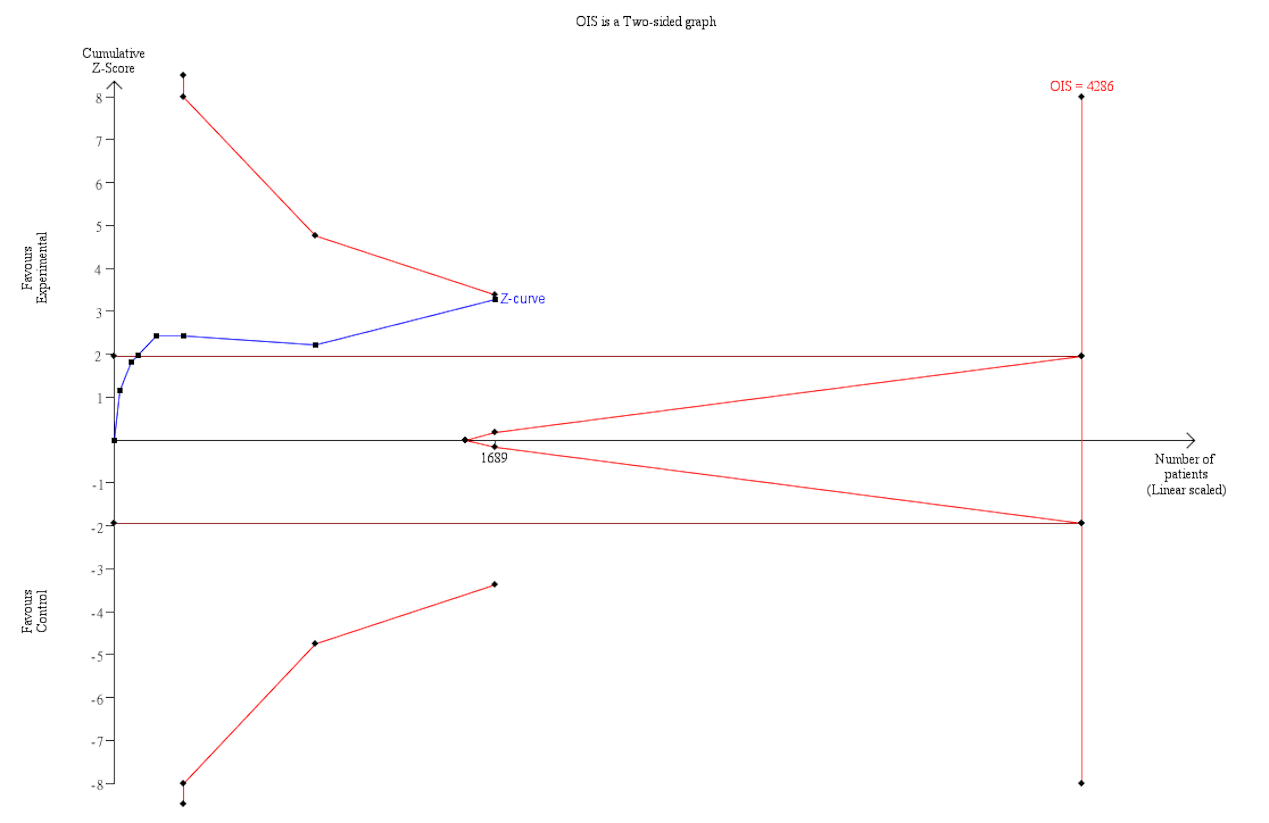


Supplemental Figure 3. Trial sequence analysis on the risk of mechanical ventilation


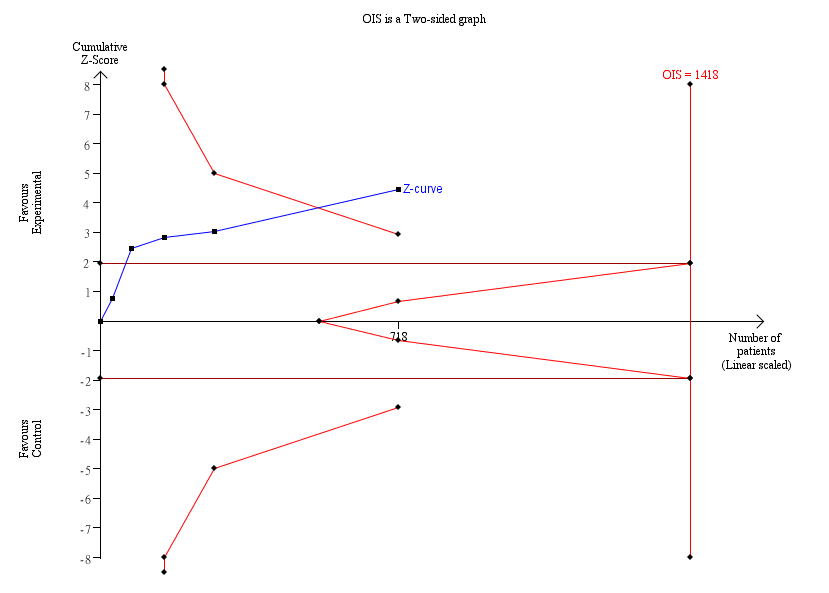


Supplemental Figure 4. Trial sequence analysis on length of intensive care unit stay


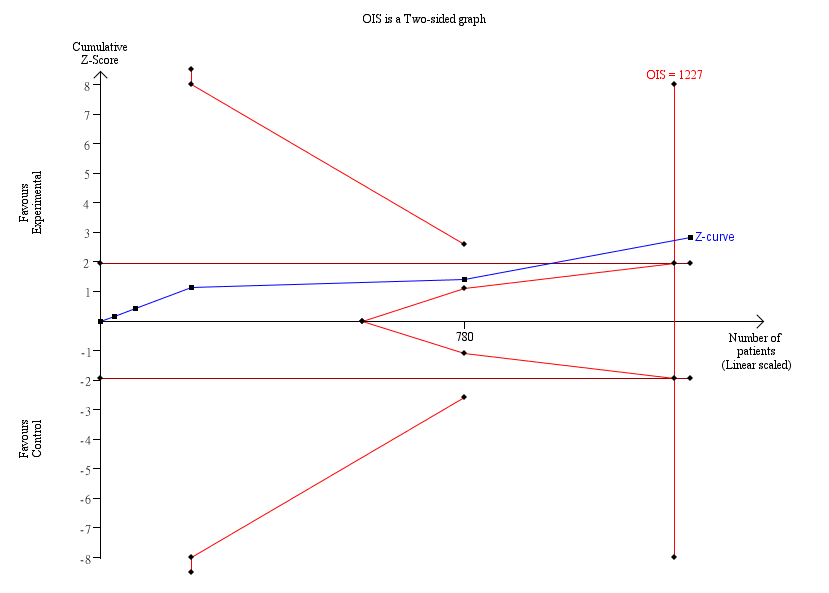


Supplemental Figure 5. Trial sequence analysis on the length of hospital stay


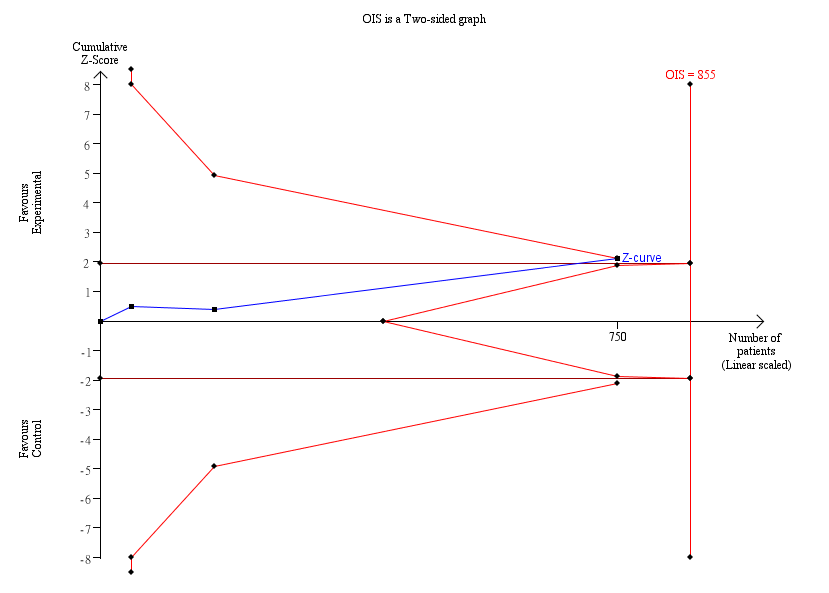


Supplemental Figure 6. Trial sequence analysis on risk of gastrointestinal tract bleeding


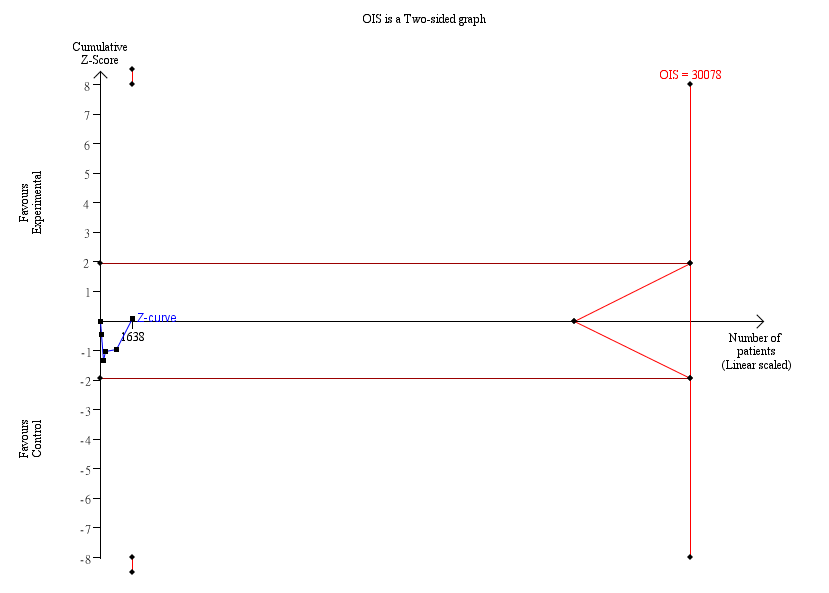


Supplemental Figure 7. Trial sequence analysis on risk of healthcare-associated infection


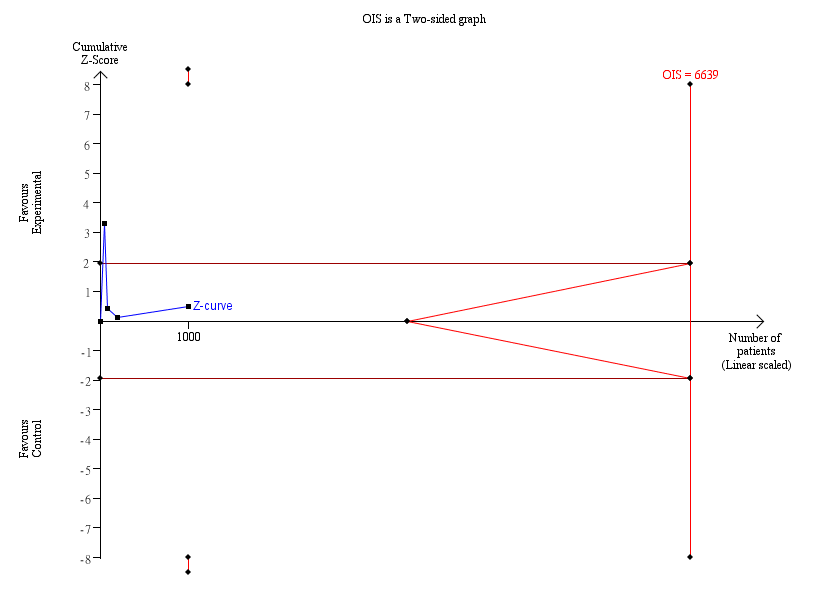


Supplemental Figure 8. Trial sequence analysis on risk of acute kidney injury


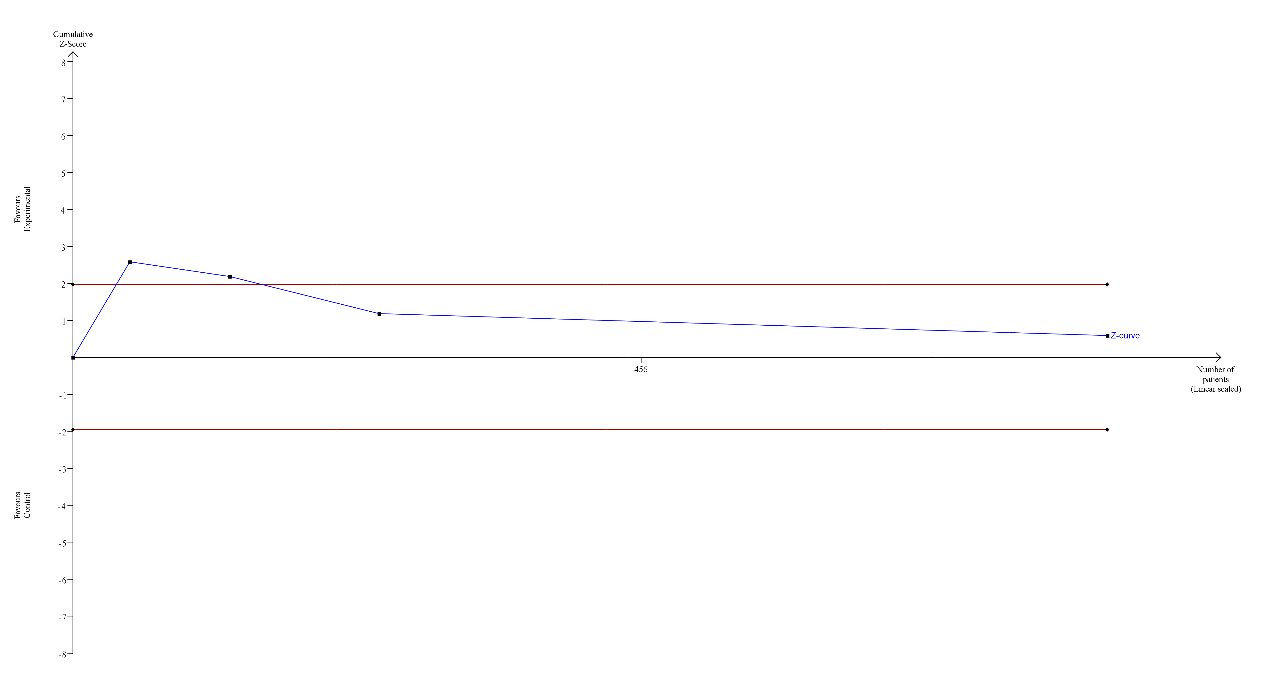

Supplement: Supplementary file 1 — Additional file 1. Figure S1 Sensitivity analysis of primary outcome by excluding each study individually. Figure S2 Trial sequence analysis on 30-day all-cause mortality. Figure S3 Trial sequence analysis on the risk of mechanical ventilation. Figure S4 Trial sequence analysis on length of intensive care unit stay. Figure S5 Trial sequence analysis on the length of hospital stay. Figure S6 Trial sequence analysis on risk of gastrointestinal tract bleeding. Figure S7 Trial sequence analysis on risk of healthcare-associated infection. Figure S8 Trial sequence analysis on risk of acute kidney injury [file 13054_2023_4561_MOESM1_ESM.docx]
